# Defining nodes in complex brain networks

**DOI:** 10.3389/fncom.2013.00169

**Published:** 2013-11-22

**Authors:** Matthew L. Stanley, Malaak N. Moussa, Brielle M. Paolini, Robert G. Lyday, Jonathan H. Burdette, Paul J. Laurienti

**Affiliations:** Laboratory for Complex Brain Networks, Department of Radiology, Wake Forest University School of MedicineWinston-Salem, NC, USA

**Keywords:** brain networks, graph theory, functional magnetic resonance imaging, neurosciences, functional connectivity, network science, neuroimaging

## Abstract

Network science holds great promise for expanding our understanding of the human brain in health, disease, development, and aging. Network analyses are quickly becoming the method of choice for analyzing functional MRI data. However, many technical issues have yet to be confronted in order to optimize results. One particular issue that remains controversial in functional brain network analyses is the definition of a network node. In functional brain networks a node represents some predefined collection of brain tissue, and an edge measures the functional connectivity between pairs of nodes. The characteristics of a node, chosen by the researcher, vary considerably in the literature. This manuscript reviews the current state of the art based on published manuscripts and highlights the strengths and weaknesses of three main methods for defining nodes. Voxel-wise networks are constructed by assigning a node to each, equally sized brain area (voxel). The fMRI time-series recorded from each voxel is then used to create the functional network. Anatomical methods utilize atlases to define the nodes based on brain structure. The fMRI time-series from all voxels within the anatomical area are averaged and subsequently used to generate the network. Functional activation methods rely on data from traditional fMRI activation studies, often from databases, to identify network nodes. Such methods identify the peaks or centers of mass from activation maps to determine the location of the nodes. Small (~10–20 millimeter diameter) spheres located at the coordinates of the activation foci are then applied to the data being used in the network analysis. The fMRI time-series from all voxels in the sphere are then averaged, and the resultant time series is used to generate the network. We attempt to clarify the discussion and move the study of complex brain networks forward. While the “correct” method to be used remains an open, possibly unsolvable question that deserves extensive debate and research, we argue that the best method available at the current time is the voxel-wise method.

## Introduction

The brain is a complex network with an underlying organizational structure. This organizational structure can be investigated using the methods of network science. The study of complex brain networks has dramatically developed and matured over the past decade, becoming the method of choice for analyzing functional brain imaging data. While network science holds great promise for expanding our knowledge of the human brain in health, disease, development, and aging, the rapid expansion and increased popularity of network science as a paradigm for analyzing neuroimaging data generates the risk that new methods may be misapplied or misinterpreted, leading to inaccurate and misleading results.

Fundamentally, all networks are composed of two basic components: the elements of the system and the pairwise relationships between those elements. Formally, graphs represent these elements as nodes and the pairwise relationships between elements as edges/links. Graph theory provides a rigorous, well-established framework for describing brain connectivity, both locally and globally, providing the first robust opportunity to expansively and non-invasively explore the entirety of the human brain at one time (Bullmore and Sporns, 2009; Rubinov and Sporns, 2010). In functional brain networks, nodes represent some predefined collection of brain tissue, and edges measure functional connectivity between pairs of nodes. Functional connectivity is an observable phenomenon quantifiable with measures of statistical dependencies, such as correlations, coherence, or transfer entropy (Friston, 1994, 2011). Once the brain network has been generated, standard network science measures can elucidate many different features, both local and global, of the interactions between brain areas.

For networks to adequately model physical systems, nodes must accurately and meaningfully represent the elements of the system (Butts, 2009). In most social, biological and technological networks, what constitutes a node and what constitutes a link is clearer and reasonably defined. For example, in studies of friendship networks, individual persons are an obvious choice for nodes, and instances of friendship between persons are an obvious choice for links; similarly, in studies of gene networks, genes are an obvious choice for nodes, and regulatory proteins are a reasonable choice for links. In brain networks each individual neuron could be represented as a node, and the edges between nodes could then be represented by synapses. While this has been done successfully in the significantly less complex *C. Elegans* (Sporns and Kötter, 2004; Towlson et al., 2013), it is not currently possible to image, record, or computationally analyze the estimated 100 billion neurons in the human brain, each one with ~7000 synapses (Drachman, 2005). Although the Human Brain Project and the BRAIN initiative are attempting to generate such a model, representing neurons as nodes and synapses as links in the human brain may not be a desirable objective. A full-scale replica of the human brain would be unlikely to produce an understandable model, considering that it is as complex as the real system. Current technology limits functional brain network analyses to nodes above the millimeter scale, meaning that many potentially interacting neurons and synapses will be represented as individual nodes in human brain networks.

The lack of a clear, obvious choice of what should represent a node in a functional brain network has resulted in the analysis of brain networks across a wide range of scales, ranging from 70-node (Wang et al., 2009) to 140,000-node whole brain networks (Eguíluz et al., 2005) and using a variety of parcellation schemes dependent on wide-ranging definitional criteria. Nevertheless, the manner in which nodes are explicitly defined in brain networks largely determines the neurobiological interpretation of the network topology (Butts, 2009). There is currently no universally accepted nodal definition, making this one of the most important unsolved problems in network analyses of neuroimaging data.

The purpose of this paper is to discuss concepts and issues surrounding node definition in functional brain network analyses. It is critical to acknowledge the fact that the optimal method for defining nodes remains an enigma. In any system as complex as the human brain, the boundaries that are used to define system elements are abstract and are typically related to the problem being addressed. In fact, the late complex systems scientist, Donella H. Meadows, noted that all boundaries are human in origin:

“… boundaries are of our own making and they can and should be reconsidered for each new discussion, problem, or purpose.” (Meadows and Wright, 2008)

Nevertheless, when generating a model of the human brain using functional brain networks, it is necessary to define individual nodes, and the boundaries of such nodes can dramatically influence the outcomes of the study (Smith et al., 2011). Of the commonly used available methods, some certainly have advantages over others, and node choice could change based on the questions being studied. By identifying motivations and problems in each commonly used approach, we attempt to clarify the discussion and move the study of complex brain networks forward. Here, after completing a comprehensive meta-analysis of the available literature, we provide a review of the most popular nodal definition schemes based on: individual voxels, anatomical atlases, and prior functional activation studies. We argue that the approach with the fewest problems currently available is the voxel-wise approach. For the types of knowledge currently being sought by most neuroscientists, the available alternatives to the voxel-wise approach have significant limitations.

In the debate over the best way to define network nodes, the various approaches available can be boiled down to one clear distinction: (1) *a priori* based determination of nodes, whether by atlases, prior literature, or some other functional imaging technique; and (2) no *a priori* based determination of nodes (i.e., making each voxel a node). As neuroscience explores the organization of brain networks, the authors of this manuscript believe that the smallest subdivisions possible (up to some point that is currently unknown) will yield the most unbiased and informative results. However, in keeping with Dr. Meadows' observation, it is important to remain open to possibilities that different boundary definitions are appropriate for different questions. Unless unequivocal evidence is found that one boundary definition is better than another, it is vital that neuroscientists remain open to others using various node definitions. Dogma should not be allowed to supersede scientific inquiry.

In short, we favor the voxel-wise approach to studies of the brain as a complex network, largely because it is not burdened by prior reductionistic brain research findings. Limiting network science studies of the brain based on findings from prior imaging techniques will limit our ability to discover new, unknown properties that are not observable using past methodologies (Telesford et al., 2011). One of the strengths of the network science approach is that it thrives on the fact that all brain regions are dependent rather than independent. This cannot be emphasized too strongly. Implementing *a priori* information will weaken the validity of the approach. We recognize that all currently available techniques are flawed, because any nodal definition scheme will contain the signal of many neurons. It is not clear which granulation level is best, so at this point we should use a method with the fewest prior assumptions and as much of the available data as possible.

## Node definitions in the literature

For our meta-analysis of the literature, studies were first identified using the PubMed database and included articles with the following search terms: “functional,” “brain” and “networks.” The search was limited to articles focused on human research and articles published between 01/01/2005—01/31/2013. This initial filter returned a total of 4847 research articles. These articles were then filtered to only include original research publications and to exclude literature reviews and commentary. Two different researchers independently filtered the remaining articles to exclude research that described functional networks generated from electroencephalography and magnetoencephalography as well as structural networks generated from diffusion weighted imaging or voxel-based morphometry. The final list included 155 studies that specifically applied principles of graph theory to functional magnetic resonance imaging (fMRI) data (see supplemental information for a complete list). Various methodologies were used to identify associations between the nodes across studies.

These studies were then categorized by the parcellation scheme each used for data analysis. Categories included analyses based on the voxel-level (32 articles), established anatomical atlases (86 articles) and previously published fMRI activation maps (20 articles). Several studies presented varied non-traditional parcellation schemes (23 articles), and a limited number implemented multiple parcellation schemes (5 articles).

In studies using a voxel-wise approach, the number of nodes varied greatly and ranged from under 5000 to as high as 140,000 nodes. In 2005, Eguiluz and colleagues published what is now known to be the first study that applied graph theory analysis to fMRI brain data. It is worth noting that this seminal contribution to neuroimaging research was not only the first of its kind but also has been referenced over 450 times. In addition to traditional voxel-wise methods, our search revealed a new variant to voxel-wise methodology. In this approach a voxel-wise based correlation matrix was generated using resting-state functional connectivity MRI (rs-fcMRI) data. The voxels were then clustered into groups using various methods to define the boundaries rather than using anatomic-based atlases (Thirion et al., 2006; Cohen et al., 2008; Mumford et al., 2010; Vejmelka and Palus, 2010; Craddock et al., 2012; Blumensath et al., 2013). These boundaries were ultimately used to create putative functional areas that are, in effect, made up of combinations of the original voxels. The purpose of this approach is to create a universal functional atlas of the human brain that can be standardized and used by researchers interested in both resting-state and task-based functional neuroimaging.

Although the initial application of graph theory principles to neuroimaging brain data was voxel-based, parcellation of the brain for functional network analysis has moved toward the use of anatomical atlases. These atlases are strictly defined using anatomical features of the brain, like locations of common gyri and do not rely on any functional information. Atlases are generally standardized, readily available, and most often used for both structural and functional neuroimaging analyses. The total number and size (i.e., voxels) of the regions that make up the entirety of the brain differs across anatomic atlases. To generate networks using an atlas-based approach, the blood-oxygen-level-dependent (BOLD) signal from all voxels is averaged within each ROI. The average time-series for all the ROIs are used to generate the final correlation matrix.

We found that the Automated Anatomical Labeling (AAL) atlas was the most commonly used, and the second most commonly used atlas was the Harvard-Oxford probabilistic atlas with a threshold between 25 and 30%. It should be noted that many studies did not use all the ROIs contained within a particular atlas. Instead, researchers picked regions that were of interest to the authors or re-sampled data to create ROIs of uniform size.

The third most common approach to parcellation was based on the findings of prior fMRI activation studies. Studies implementing this approach used significant clusters found within group activation maps that were previously published by the authors themselves or by others who had published findings pertinent to a paper's topic. In addition to this, others performed a meta-analysis of several different resting-state or task-based fMRI activation maps to define a parcellation. For all these studies, a Gaussian kernel with a full-width-half maximum of 3–10 mm centered on the peak coordinates was used to create ROIs, and in all instances this approach excluded much of the brain available for analysis.

### Voxel-wise approach

The approach we favor for defining nodes in functional brain networks treats each individual voxel as a node. Voxel-wise networks are constructed by assigning a node to each, equally sized brain area (voxel), and then measuring the relatedness in activity computed from pairs of simultaneously recorded time series. The voxels are based on a grid placed on the brain during imaging and then warped to MNI (Montreal Neurological Institute) standard space during the image preprocessing. The placement is arbitrary, but voxels are approximately aligned across subjects during the warping procedure. It is acknowledged that perfect alignment is not achieved, but interpretations of the results really should never come down to a single voxel.

Among those who utilize a voxel-wise approach, the number of nodes varies widely. Voxel-wise approaches range from incorporating roughly 3400 nodes (Liu et al., 2011) to 140,000 nodes (Eguíluz et al., 2005) in the brain network. This variability is due to differences in acquisition resolution and to researchers choosing to limit their analyses to the voxels in specific regions. Such differences in network size will produce differences in network metrics, as is the case for all three major approaches for defining nodes. This will make comparison between quantified variables from voxel-wise networks of different sizes difficult to interpret. However, if the entire brain is included and the density of the connections is controlled, then comparisons across studies with different-sized networks are possible. The size of the network should not significantly interfere with comparisons of the location of key nodes within the brain.

#### Discussion

A common criticism of the voxel-wise approach is that connectivity between neighboring nodes is spurious and over-represented. This is because local spatial correlations due to many non-neural factors may manifest as edges even though there may not be direct functional connectivity (Power et al., 2011). One possible cause of increased local correlations is the result of reslicing and blurring in data processing (Wig et al., 2011). Because reslicing and blurring are inevitable steps in standard data processing, it is only possible to partially alleviate the effects of voxels sharing non-biological signal. Another possible cause is that the fMRI signal is actually due to changes in blood flow, and local voxels share local blood flow. Thus, changes in regional blood flow could increase local correlations even if there are not strong associations in neural activity. However, it is important to note that the neurobiology is such that neurons are, in fact, mostly connected to nearby neurons. The probability of connection and the number of connects falls off following either a Gaussian or exponential process. As such, the probability of connection and number of contacts drops dramatically beyond 0.5 mm from the neuron cell body (Liley and Wright, 1994; Hellwig, 2000). Sporns and Zwi (2004) further demonstrated that a model containing predominantly local connections with sparse distant connections best captures small world topology in an anatomical brain network. So, even though blood flow may be locally coupled, so are real neurons. Therefore, it is difficult to know how much is an artifact of fMRI and how much is real due to the fact that most connections are local.

To examine potential effects of local correlations on network metrics, Hayasaka and Laurienti (2010) deleted local edges connecting spatially neighboring voxels in the voxel-wise network in subjects and then recalculated network metrics. They examined network metrics on networks that were derived by applying various thresholds to the correlation matrix that produced networks of different edge densities. They found reduced clustering and increased path length due to the deletion of local edges. In brief, clustering is a measure of the connections that exist between the neighbors of a given node, whereas the path length is a measure of the number of steps required to get from one node to another node (for further detail, see Watts and Strogatz, 1998). The effect was minimal when the density of edges in the network was set such that greater than 98% of the voxels remained connected to the network. Large changes in clustering and path length did occur when substantial portions of the network became fragmented. The degree distribution did not change dramatically despite the deletion of local edges. In order to avoid adding spurious local correlations to the network as much as possible, data should not be spatially smoothed. Smoothing introduces spuriously high correlations between adjacent voxels. Even if no spatial smoothing is performed in the preprocessing protocol, fMRI data inherently contains some degree of spatial smoothing due to the data acquisition process. For example, the spatial normalization process itself could introduce local correlations, which could bias the structure of the network. This incidental spatial smoothing can produce specious short-range connections that in turn inflate the clustering metrics in high-resolution voxel-wise networks (van den Heuvel et al., 2008). It should be noted that this is less of a concern for lower-resolution networks, because the size of each node greatly exceeds the spatial extent of the smoothing range (Zalesky et al., 2012). Another criticism of the voxel-wise approach is that relationships between short distance nodes are especially susceptible to spurious augmentation by subject motion (Power et al., 2011). However, subject motion is not merely restricted to local voxels, as the head moves as a rigid body. As such, most motion artifacts are associated with the interface of different brain tissue types (e.g., gray matter/white matter interface) (Field et al., 2000), and regions across hemispheres can become correlated because of motion (Bright and Murphy, 2013) rendering this a problem for any parcellation scheme.

Another criticism of the voxel-wise approach is that there are serious signal-to-noise (SNR) problems and spurious connections due to the low signal in the small voxels. In particular, it has been suggested that adopting an excessively high spatial resolution may be associated with a disproportionate loss in SNR (Fornito et al., 2010). This will result in an increase in the random connections in the network. It essentially will be like adding random links to the true network. This would have the effect of decreasing the overall network path length, but it would likely not affect the local clustering (Watts and Strogatz, 1998). The main opponents of the voxel-wise approach argue that the problem is an increase in clustering, not a decrease in the path length (Power et al., 2011). If the SNR of voxel-wise data were not of sufficient quality, all traditional functional connectivity studies (e.g., Biswal et al., 1995, 1997) that serve as the foundation for network analyses in the brain would be called into question. In addition, all traditional fMRI activation studies are voxel-wise, and thus those works would be called into question.

There is no doubt that averaging signal across local voxels, as is performed with the non-voxel-wise approaches, will decrease the noise levels in each network node. In fact, we have evaluated the magnitude of the correlation values in a voxel-wise and atlas-based network averaged over 10 young, healthy subjects at rest using recently published data (Peiffer et al., 2009; Hayasaka and Laurienti, 2010). The positive correlation values were significantly higher (*p* = 0.001, paired *t*-test) when the time courses were averaged across anatomical ROIs (average *r* = 0.221, *SD* = 0.017) than when individual voxels were used (average *r* = 0.159, *SD* = 0.011). While it is true that averaging voxels reduces noise, it is also true that real signals are lost if voxels with very different true signals are averaged. This is in fact what we found when we examined the correlation values of the strong associations that are typically retained after thresholding functional networks. The thresholds were set such that the density of connetions was comparable across the types of networks. For the atlas-based network the retained edges had an average *r*-value of 0.421 (*SD* = 0.047). For the voxel-wise network, the retained edges were significantly higher (*p* < 0.001, paired *t*-test) with an average *r*-value of 0.55 (*SD* = 0.039). Thus, the averaging procedure used when combining many voxels into a single node did reduce overall noise, but it also reduced signal in the strongest of the network connections.

Despite some potential limitations, voxel-wise networks have strengths that make them an ideal choice for making new discoveries about human brain function. Representing nodes as equally sized voxels allows the voxel-wise approach to escape three serious problems facing other methods. First, voxel-wise networks are not constrained by the assumption that voxels from the same anatomical regions or functional areas are sufficiently similar so that they can be averaged to form a larger node. Second, because each node in the voxel-wise network is of equal size, signal variance will not scale with the number of voxels that contribute to its estimate, meaning that the quantification of pairwise relationships will not be disproportionately more reliable for larger brain areas (Hayasaka and Laurienti, 2010). Third, those ROIs comprised of a greater quantity of voxels than other ROIs in region-based networks may exhibit differential connectivity simply due to the fact that a greater variety of signals are included in the ROI itself (Hayasaka and Laurienti, 2010). Because each node is the same size in voxel-wise networks, no correction mechanism need be developed to account for differences in the spatial extent of ROIs.

The voxel-wise approach generates high-resolution (mesoscopic) brain networks, allowing researchers to acknowledge and account for (given current technological constraints) the heterogeneity of areas present within the larger ROIs identified by other parcellation techniques. For instance, in a recent study a highly interconnected hub in the posterior cingulate cortex (PCC) observed in a high-resolution voxel-wise network was centered in the middle of three different ROIs (nodes) in a network with nodes defined with an AAL atlas (Hayasaka and Laurienti, 2010). Regardless of whether the three adjoining ROIs in the atlas-based network were kept separate or averaged together, it would not have been possible to meaningfully capture the high degree area in the middle of the three anatomical ROIs. Additionally, although both anatomical atlas-based and voxel-wise network analyses have consistently identified the PCC and the nearby precuneus as highly connected nodes, or hubs (Hagmann et al., 2008; van den Heuvel et al., 2008; Buckner et al., 2009), only voxel-wise networks allow for the precise localization of hub nodes within these anatomical areas (Hayasaka and Laurienti, 2010). It should be noted that (Tohka et al., 2012) compared a voxel-wise (40,962 nodes) and an anatomical atlas-based (54 nodes) network with results corroborating those of Hayasaka and Laurienti (2010). This ability to more accurately identify the spatial locations of hubs in functional brain networks allows researchers to more accurately quantify the assortativity of the network, efficiency in the flow of information in the network, resiliency of the network to targeted and random attack, and the nature of the degree distribution of the network (whether the degree distribution is truly scale-free or instead an exponentially truncated power law degree distribution). For overviews of these metrics, see Bullmore and Sporns (2009), Rubinov and Sporns (2010), and Kaiser (2011). Additionally, a substantial amount of research has indicated that hubs are radically reorganized in a variety of neurological disorders, including Alzheimer's disease (Supekar et al., 2008), stroke (Desmurget et al., 2007), schizophrenia (Lynall et al., 2010) and abnormalities in consciousness (Achard et al., 2012). This suggests that the ability to accurately and effectively detect the location of hubs may serve an important purpose in clinical settings.

Several extensions of the voxel-wise approach have been developed using rs-fcMRI correlations by attempting to group together voxels with similar properties. Each group of voxels, or functional “unit,” can potentially represent a node for further network analyses. The full set of fundamental units of interest can, in theory, be described with a robust brain map akin to a map of the countries of the world, wherein each country is analogous to a distinct functional unit (Wig et al., 2011). Methods used to identify functional units (nodes) using rs-fcMRI data can be classified into 3 categories: detecting sharp transitions in rs-fcMRI patterns (Cohen et al., 2008; Barnes et al., 2010, 2012; Nelson et al., 2010), identifying functionally similar clusters (Thirion et al., 2006; Mumford et al., 2010; Vejmelka and Palus, 2010; Craddock et al., 2012), and region growing methods (Blumensath et al., 2013).

Some who have advocated for these rs-fcMRI approaches and against a voxel-wise approach argue that although interrogating voxels is suitable in the statistical analysis of neuroimaging data when the goal is to identify groups of voxels with similar properties, treating a voxel as a node in a network explicitly implies that it is being modeled as a distinct unit of information processing (Wig et al., 2011). The implication here is that because voxels are not distinct units of information processing, voxels should not represent nodes for network analysis. We contend that such an argument is circular. The techniques that have been developed using rs-fcMRI data are built upon what is fundamentally a voxel-wise network analysis. In fact, (Barnes et al., 2010), who implement an algorithm to detect transitions in connectivity patterns to form boundaries between nodes, admit that each voxel is treated as a node and the similarity measure (i.e., η^2^) between nodes is treated as an edge in order to then find sharp transitions in connectivity. Similarly, clustering and region-growing methods initially require the detection of correlations between adjacent voxels in order to then determine whether those voxels should be grouped together. Groups of voxels identified by each respective approach are redefined as a single node, so that researchers can go back to the original data and perform a new network analysis using node definitions that were based on a voxel-wise network analysis. If one has problems with a voxel-wise analysis, then it is unreasonable to use a voxel-wise analysis to define a nodal set in an attempt to avoid a voxel-wise analysis. It is necessary to represent each voxel as a unit of information processing when identifying putative functional units, which then become the new units of information processing. Importantly, because each approach is derived from a voxel-wise network analysis, the problems previously presented for the voxel-wise approach are conferred upon those methods developed using rs-fcMRI data.

The optimal method for combining voxels into functional “units” remains an enigma. This is largely due to the inherent inability to access any form of ground truth indicating that a given method successfully parcellates functional units in the brain (Craddock et al., 2012; Lohmann et al., 2013). A common strategy has been to identify algorithms that yield results comparable to past methodologies. However, this strategy will bias results toward prior traditional fMRI research and hamper the ability to make new discoveries. Others check for reproducibility of results generated by some new algorithm. However, the ability to accurately reproduce results does not entail that an algorithm has successfully delineated the “true” set of functional units across the brain. The fact that some approach is reproducible does not mean than it is accurate; it merely means that it is reproducible. A better approach to validating new parcellation schemes would be to identify which methods best predict different behaviors or pathologies through extensive research. Until there is evidence that unequivocally demonstrates that a true whole-brain functional parcellation exists, we contend that the least *a priori* information that is included, the better.

### Structural anatomical atlases

The most widely used parcellation scheme defines nodes as individually segregated anatomical regions-of-interest (ROIs) from one of the many readily available structural anatomical atlases. The mean time series is estimated for every subject by averaging the fMRI signal over all voxels in each anatomically defined ROI. Of the many modern structural brain atlases readily available, the number of ROIs (nodes) typically ranges from 70 to 250. The most widely used anatomical atlas in functional brain network studies is the standard AAL template, which parcellates the cortex and subcortical structures by identifying gyral and sulcal boundaries. The full AAL template comprises 116 ROIs (nodes) (Tzourio-Mazoyer et al., 2002), but the cerebellum is often omitted in network-based studies (Zalesky et al., 2010b) leaving the cerebral hemispheres divided into 45 anatomical regions each. In our literature search the AAL atlas was used to define nodes in 69 of the 86 total studies that used structural anatomical atlases. However, it should be noted that some studies have used at least two different atlases in network analysis, one of which being the AAL atlas.

In addition to the variability in choosing either the full 116 node network or a partial set of 90 nodes, it is not uncommon for researchers (1) to only select particular regions from the AAL atlas considered *a priori* to be of particular interest based on preexisting literature (Çiftçi, 2011), (2) to exclude ROIs with less than some predetermined percentage of brain coverage (Fornito et al., 2011), or (3) to subdivide the AAL atlas into a greater quantity of nodes of roughly homogenous size constrained to lie within the volume encapsulated by its parent AAL ROI (Fornito et al., 2010; Zalesky et al., 2010a; Zhang et al., 2011; Achard et al., 2012). Among those who use the AAL atlas, variability in the number of nodes ranges from a partial brain network of 32 nodes (Çiftçi, 2011) to a whole-brain downsampled network of 4320 nodes (Fornito et al., 2010).

Similar patterns of variability in parcellation occur when different researchers use other structural anatomical atlases. For instance, among those who use the Harvard-Oxford probabilistic atlas, it is not uncommon (1) to use the full set of ROIs covering 48 cortical and 21 subcortical structural areas corresponding to portions of cortical gyri and subcortical gray matter nuclei, (2) to select only particular ROIs from the atlas considered a priori to be of particular interest based on preexisting literature (Lord et al., 2011), (3) to entirely exclude data from certain ROIs due to suboptimal registration (Davis et al., 2013), or (4) to subparcellate the atlas into a greater quantity of regions of roughly uniform size entirely contained within a single parent ROI from the original atlas (Alexander-Bloch et al., 2012, 2013). Among those who use the Harvard-Oxford probabilistic atlas, variability in the number of nodes ranges from a partial network of 19 nodes (Lord et al., 2011) to a whole-brain downsampled network of roughly 300 nodes before thresholding (Alexander-Bloch et al., 2012). Our literature search revealed that 10 studies have used the Harvard-Oxford Probabilistic Atlas to define network nodes.

#### Discussion

Proponents of using anatomical atlases to define nodes for network-based fMRI studies often argue that ROIs should represent areas with clear anatomical boundaries in order to preserve the interpretability of results from functional connectivity studies. Others argue that it is only possible to establish relationships between brain structures and their functions by defining nodes based on anatomical features (Tzourio-Mazoyer et al., 2002). Despite the variability in the quantity of nodes and percentage of brain space used in the literature, the fact that the size and extent of each node remains fixed within a single atlas across subjects and studies is an advantage for the approach. The lack of variability in this regard has the potential to neatly standardize studies, allowing for the meaningful comparison of results across studies.

However, the various anatomical brain atlases currently available exhibit remarkable differences in the number, shape, and location of ROIs. Because the properties of ROIs are highly non-linear, a slight change in the number, shape, or location of ROIs can dramatically alter connectivity profiles (Li et al., 2010), producing profound effects on network metrics. Low spatial resolution anatomical templates are more likely to combine areas that have distinct temporal signals (Fornito et al., 2010; Craddock et al., 2012). Averaging disparate temporal signals will decrease the signal-to-noise because the signals of interest are actually averaged out, thus adding noise to the network analyses. In fact, significant differences in network metrics have been observed across different anatomical templates. For instance, Wang et al. (2009) examined statistical differences in the topological properties of functional brain networks between an AAL network (90 node) and an Automatic Non-linear Imaging Matching and Anatomical Labeling (ANIMAL) network (70 node). While both networks exhibit robust small-world attributes and an exponentially truncated power law degree distribution, the majority of other local and global topological parameters vary significantly across the two networks. Further, because of significant differences in both the quantity of nodes and the percentage of brain space incorporated in network analyses among researchers who use variants of the same anatomical atlas, meaningfully comparing results between any two studies is potentially difficult, impractical, and misleading (Honey et al., 2009; Kaiser, 2011).

Because of the coarse resolution of anatomical atlas-based approaches to defining nodes, these atlases are most likely to collapse many different, interacting brain areas with different functions into a single node. Consequently, representing many different interacting groups of neurons (and synapses) with different properties as single nodes may poorly represent reality, obscuring the differences between smaller units within the collapsed node. The regions defined by anatomical atlases should not be expected to contain homogenous functional connectivity for two distinct reasons. First, because ROIs derived from most atlases are so large, it is more likely that they include signals from several different functional sub-regions (Hayasaka and Laurienti, 2010). To overcome this problem, some researchers (e.g., Hagmann et al., 2008; Achard et al., 2012) have randomly subdivided ROIs, mitigating the possibility of mixing BOLD time series. While randomly subdividing anatomical ROIs has been shown to increase regional homogeneity (Craddock et al., 2012), there is no reason to assume that these randomly subdivided parcels accurately represent structural patterns in the underlying neuroanatomy. Second, and more importantly, putative functional areas simply do not need to obey the divisions created by any parcellation scheme based on anatomical landmarks. Putative functional areas could extend across a morphological boundary, or multiple putative functional areas may be present within a single morphologically defined parcel. Changes in state or condition could also result in changes in the organization of putative functional areas, which need not map onto divisions based on anatomical features.

**Figure 1:**
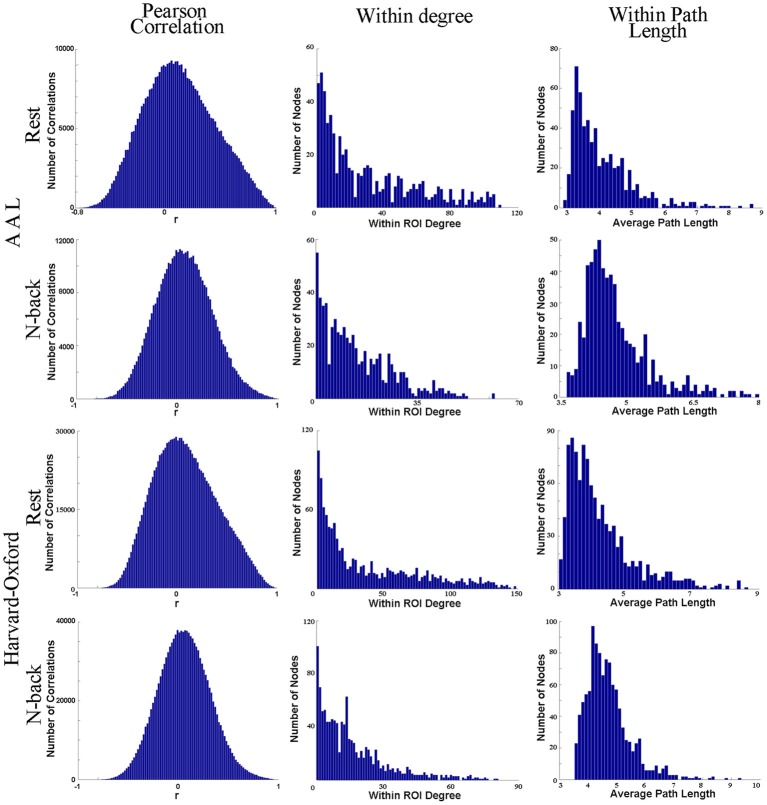
**This figure shows one anatomical ROI (node) from the AAL atlas (left precuneus) and another from the Harvard-Oxford Probabilistic Atlas (precuneus, as the atlas does not separate the precuneus into two separate ROIs).** Column 1 shows four histograms, each depicting the correlation of the time series of each voxel with every other voxel within the identified anatomical ROI in a single representative subject at rest and while engaged in a standard 2-back task. For each histogram depicting each ROI identified from each atlas and each condition, the voxels within the identified ROI (node) are poorly correlated with one another overall. This suggests that the *SNR* ratio is low. In the second and third columns, the degree distribution and average path length have been plotted for those voxels within each anatomical ROI exclusively with respect to all other voxels within the ROI. We have excluded connections to other voxels outside of the ROI. Presumably, if the identified functional area were functionally homogenous, then at best we would see every single voxel within the region connected (strongly positively correlated) to every other voxel within the region, or at worst, we would see the degree distribution following a normal curve. However, column 2 shows that (1) not every voxel is connected to every other voxel in the putative ROI and that (2) the degree distribution within the ROI itself follows a power-law instead of a uniform distribution. A significant sum of voxels within each ROI has relatively few connections (strong positive correlations) to other voxels within the ROI, and some have zero connections to other voxels within the ROI itself. No voxel within either anatomical ROI has an average path length of one within the ROI itself ((Wig et al., 2011) claim that each voxel must be connected to every other voxel within an ROI in order for the ROI to be a functional unit). Consequently, different subregions within the ROIs likely have different topological features, and consequently contribute to the structure and function of the network as a whole in different ways.

In fact, anatomical atlases have been shown to exhibit poor ROI homogeneity, failing to accurately reproduce functional connectivity patterns present at the voxel scale (Craddock et al., 2012). To further demonstrate this point, we have randomly chosen one anatomical ROI (node) from the AAL atlas (left precuneus) and another from the Harvard-Oxford Probabilistic Atlas (precuneus, as the atlas does not separate the precuneus into two separate ROIs). Using recently published data (Rzucidlo et al., 2013), we randomly selected a subject (25 year old, healthy female). Data were acquired at 4 mm × 4 mm × 5 mm voxel-size, and each voxel was represented as a node for network analysis. We employed Pearson's correlation coefficient (a commonly used correlation measure in the literature) to compare the time series of each voxel with every other voxel within the identified anatomical ROI in a single representative subject at rest and while engaged in a standard 2-back task. To generate networks of comparable edge density, the resting state data were thresholded at 0.6996 and the 2-back data at 0.6520, in accordance with the thresholding strategy laid out in Hayasaka and Laurienti (2010). Figure 1 demonstrates that the voxels within the identified ROI are poorly correlated (the average *r*-value is close to 0) with one another within both the AAL atlas ROI or the Harvard-Oxford probabilistic atlas ROI. If these were homogeneous regions (i.e., the voxels/brain regions are “behaving the same”), then the time series of the voxels within the ROI would be very similar, yielding high *r*-values.

We have also plotted the degree distribution and average path length for those voxels within each anatomical ROI exclusively with respect to all other voxels within the ROI. Figure 1 demonstrates that the voxels within each anatomical ROI do not have a normal degree distribution, as one would expect should each ROI be functionally homogenous. Instead, a significant quantity of voxels within each ROI has a very small number of connections to other voxels within the ROI. Additionally, because no voxel within either anatomical ROI has an average path length of one within the ROI itself, different subregions within the ROIs have different network properties.

### Functional activation meta-analytic approaches

Another approach to defining nodes in functional brain networks utilizes results from preexisting traditional task-evoked fMRI studies to identify a set of fixed ROIs for all subsequent studies. Some ROIs have been chosen from prior task-evoked fMRI studies examining either an individual cognitive function or a limited set of cognitive functions, which are then implemented in subsequent network analyses (e.g., Fair et al., 2007; Nomura et al., 2010; Rish et al., 2013), while others have used data derived from a diverse set of studies and performed a meta-analysis to define a set of nodes to be used for any future network analysis (Power et al., 2011; Wang et al., 2011). Among those who use preexisting data from a diverse set of task-evoked fMRI studies, some further include resting-state functional connectivity MRI (rs-fcMRI) data to locate additional ROIs to include in the set of nodes (Power et al., 2011).

Nodes defined using fMRI activation data are invariably, though not necessarily, modeled by spheres typically of 3–6 mm radii, fixed on either points of peak activity within a putative functional area (Power et al., 2011; Stevens et al., 2012) or center of mass coordinates of an putative functional area (Dosenbach et al., 2007). These differences in sphere radii across studies translate to dramatic differences in volumes ranging from roughly 113–905 mm^3^. Incorporating spheres to define functional areas excludes all voxels except those in the spheres. The spheres are meant to represent all activity within putative functional areas. This is thought to minimize the likelihood of crossing the boundaries of a functional area. Using spheres does invalidate concerns related to the problems of signal variance scaling and the differences in the number of ROI connections due differences in ROI sizes that affect anatomical atlas-based networks (Wig et al., 2011). In the available literature no study has utilized a set of spherical ROIs covering more than 25% of the cerebral cortex and subcortical nuclei; many only cover a fraction of 1% of the cerebral cortex and subcortical nuclei (e.g., Nomura et al., 2010; Rish et al., 2013). The number of nodes in studies utilizing this parcellation strategy range from roughly 10–264 nodes.

#### Discussion

One of the primary motivations for limiting the functional activation-based method to spheres is predicated upon the idea that the voxel-wise approach is hampered by a tendency for nearby voxels to share non-biological signal (causing increased functional connectivity correlation) and that short-distance relationships are especially susceptible to spurious augmentation by subject motion (Power et al., 2011; Wig et al., 2011). However, these same concerns may be problematic for functional activation approaches as well, because each sphere is composed of a set of neighboring voxels. If such artifacts are indeed predominantly local, averaging signal from adjacent voxels will not average out these signals because the spurious signals are located in the adjacent voxels that are being averaged.

Proponents of this approach often argue that by using an extensive meta-analytic procedure, it is possible to accurately identify discrete functional macroscopic “units” of brain organization, each of which representing a distinct unit of information processing. These macroscopic “units” supposedly best represent well-formed nodes in network analyses. This contrasts with the voxel-wise approach wherein voxels are not meant to correspond to macroscopic units of brain organization. Consequently, proponents of the meta-analytic approach argue that there is no reason to believe that a voxel-wise approach incorporates well-formed nodes (Power et al., 2011; ?). Operating under this presupposition, proponents of the meta-analytic approach argue that the failure of the voxel-wise approach to properly model macroscopic functional “units” has practical implications that will distort network measures. The argument runs as follows:

“As all voxels existing within a functional area undoubtedly share an edge with one another, graph measures that focus on specific properties of nodes will be biased toward nodes (voxels) existing within areas (and possibly communities) that are larger than others, and measures describing global properties of the graph will be distorted due to a misrepresentation of areas as a function of the number of voxels they contain” (Wig et al., 2011).

We contend that this criticism is unfounded, and it is simple to demonstrate that all voxels existing within functional areas do not share an edge with one another, assuming that a reasonable network density is used (obviously, if no threshold is applied and all nodes are connected to all nodes, then the statement above would be true).

**Figure 2:**
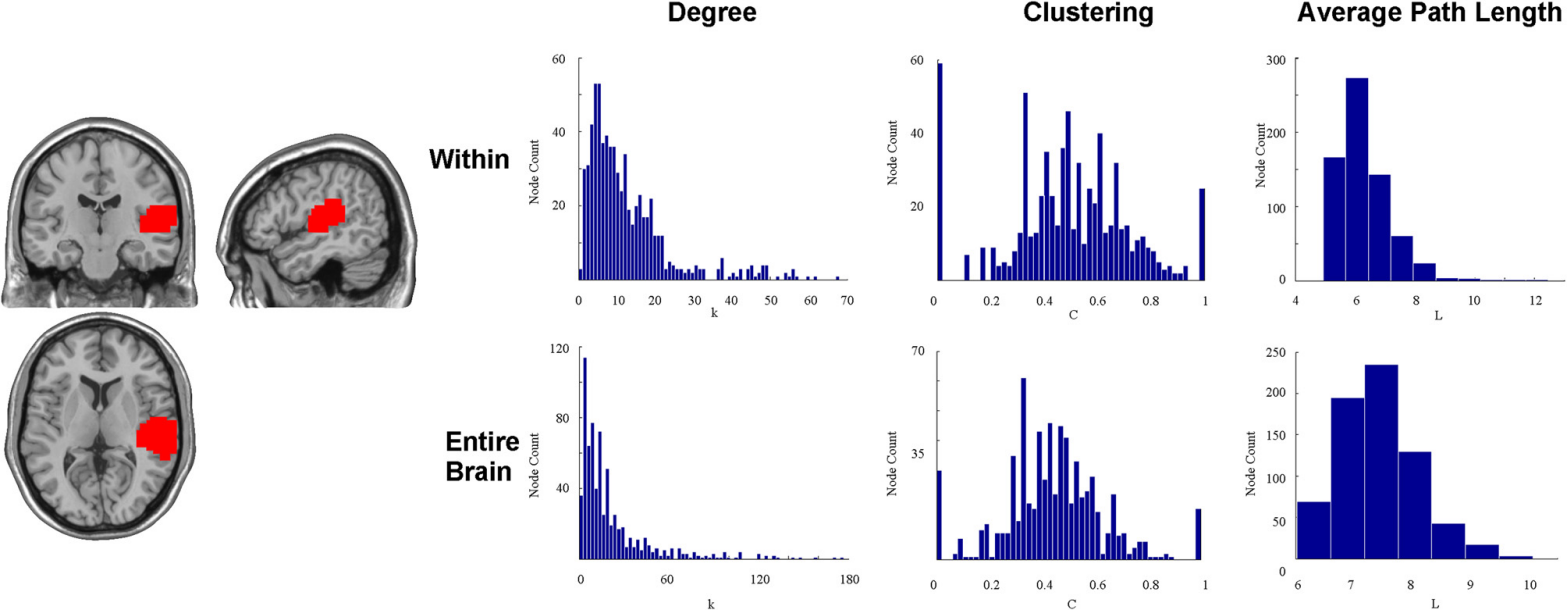
**This figure shows an auditory ROI (*T* > 10) identified in a previous activation study during which subjects were exposed to 2-Hz bursts of white noise alternated with silence.** Subjects had to identify a 500-Hz tone randomly embedded in the white noise. Presumably, if the identified functional area were functionally homogenous, then at best we would see every single voxel within the region connected to every other voxel within the region, or at worst we would see the degree distribution following a normal curve. However, This figure shows that not every voxel is connected to every other voxel in the putative ROI, and the degree distribution within the ROI itself is not a uniform distribution. Instead, a significant quantity of voxels within each ROI has relatively few connections to other voxels within the ROI, and the degree distribution follows a power law distribution instead of a normal distribution. Additionally, if the ROI were functionally homogenous, then clustering should be close to a value of one for each and every node within the region. However, This figure shows that this is not the case. Instead, there is a wide distribution of clustering values for those voxels contained within the ROI. Finally, if the ROI were functionally homogenous, then average path length should be close to one (or precisely one) for all voxels within the ROI. However, This figure shows that it often requires many steps to get from one voxel to another within the ROI. This figure also shows the degree distribution, path length, and clustering for all voxels within the identified ROI, but without discarding all other voxels outside of the putative functional area. It is clear that different subunits (voxels) within the putative auditory ROI may serve very different functions with respect to global topology of the network.

To illustrate this point, we attempted to reproduce a portion of this method by examining a spatially contiguous auditory ROI in the right cerebral hemisphere identified in a previous task-evoked fMRI analysis (Peiffer et al., 2009) as used in Figure 2. A total of 61 normal, healthy adults ranging in age from 18 to 80 years old were included. In the task portion of the auditory paradigm, 2-Hz bursts of white noise alternated with silence. Subjects had to identify a 500-Hz tone randomly embedded in the white noise. All participants were included in a random-effects group analysis and the activation map was thresholded at a t-score greater than 10 (one sample, one tail, *t*-test with 60 *df*). The region of activation in the right auditory cortex was then used as the region of investigation. A resting-state brain network was then created at the voxel level using a random individual study participant (healthy 25 year old female) from a different study (Rzucidlo et al., 2013). The connectivity profile of the auditory area was then explored. The degree distribution, path length, and clustering for all voxels, exclusively within the identified ROI by disregarding all other voxels outside of the putative functional area, are shown in Figure 2. Presumably, if the identified functional area were functionally homogenous, then at best we would see every single voxel within the region connected to every other voxel within the region, or at worst, we would see the degree distribution following a normal distribution curve. However, Figure 2 shows that not every voxel is connected to every other voxel in the putative ROI, and the degree distribution within the ROI itself is not a uniform distribution. Instead, it follows a truncated power law. Additionally, if the ROI were functionally homogenous, then clustering should be close to a value of one for each and every node within the region. However, Figure 2 shows that this is not the case. Instead, there is a wide distribution of clustering values for those voxels contained within the ROI. Finally, if the ROI were functionally homogenous, then average path length should be close to one (or precisely one) for all voxels within the ROI as Wig et al. (2011) have suggested. However, the data demonstrates that it often requires many steps to get from one voxel to another within the ROI.

To further demonstrate the heterogeneity of the voxels within this area, we calculated the degree distribution, path length, and clustering for all voxels with the entire voxel-wise brain network (Figure 2). The results show a tremendous diversity of connectivity across the voxels within this putative functional area. Such diversity may be critical for very different functions with respect to global topology of the network. In fact, many voxels within identified ROIs have shorter path lengths to voxels outside of the ROI than to those within the ROI.

**Figure 3:**
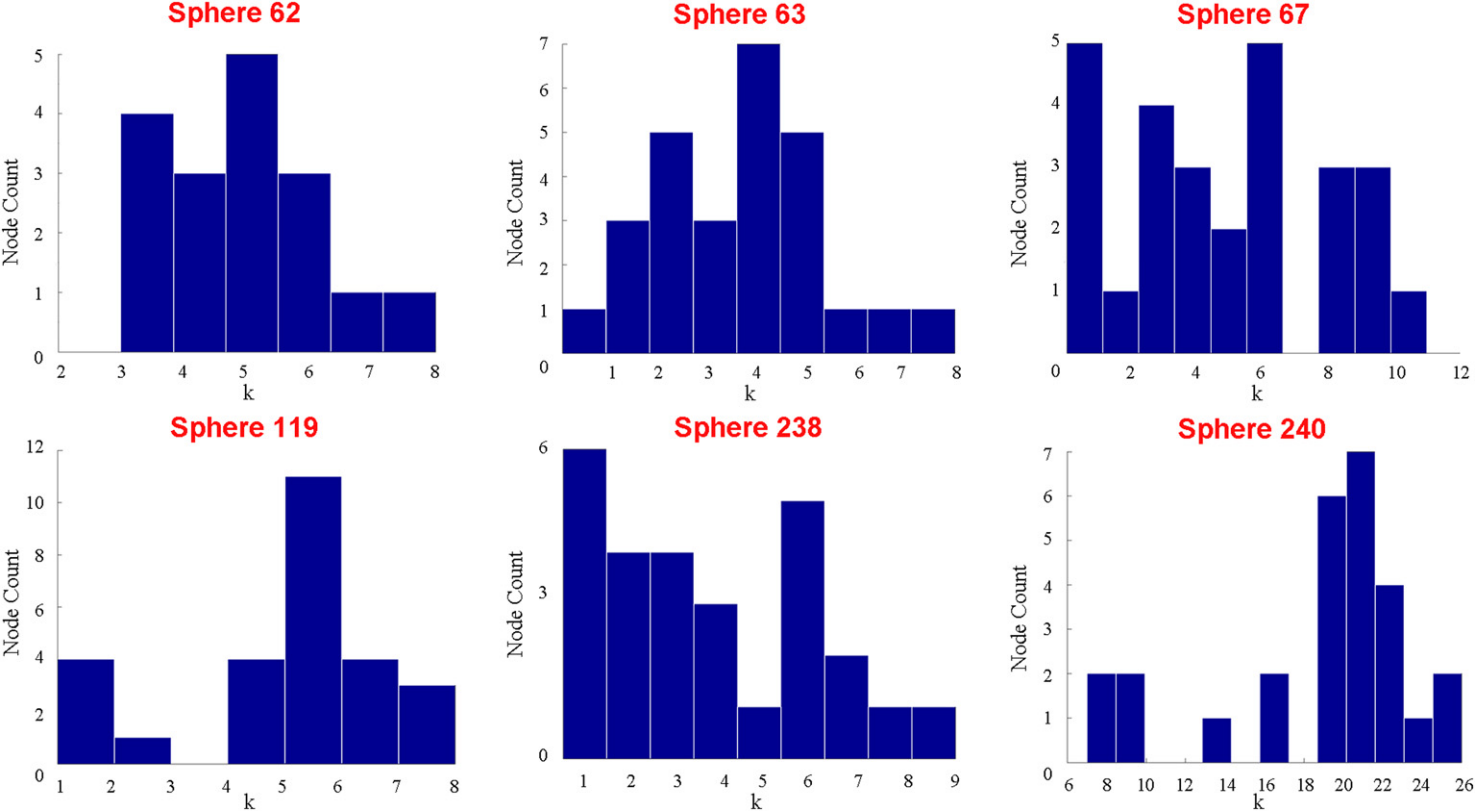
**This figure shows the respective degree distributions of 6 different cubes composed of 27 voxels each on the coordinates provided by (Power et al., 2011).** The size of each cube is smaller than the typical sphere in the literature, so if all the voxels in each sphere do not have very similar, high degrees within the sphere, then it is unreasonable to use any spheres generally to represent functional areas. Despite the fact that 27 voxels are contained within each sphere, 5 of the 6 spheres do not have more than a maximum degree of 10. Additionally, in 5 of the 6 spheres, there is at least one voxel that is not connected (highly correlated) with any other voxels in the sphere.

Those who use a meta-analytic approach might respond that the peak activity coordinates represented by the sphere itself adequately capture the properties of the putative functional area. Thus, it would only be important that the spheres are functionally homogenous. While it is clear that the auditory ROI is not functionally homogenous, and thus, the meta-analytic method fails in this regard, it is also possible to show that not even the spheres themselves are functionally homogenous. Using the same data, we randomly selected six different coordinates identified by Power et al. (2011) as peak activity locations of putative functional areas located within the auditory ROI. Nodes were represented as cubes of 9-mm length (a relatively small size among studies that use cubes or spheres), and a total of 27 voxels were contained within each node. Figure 3 shows the degree distribution of all voxels within each of the cubes. No voxels in any of the six cubes share an edge with all other voxels within the cube. In fact, across all the cubes, there are only 2 voxels that have connections with 20 of the possible 26 other voxels. Most voxels have 10 or fewer connections with the potential 26 other voxels. Consequently, it is not true that “all voxels within a functional area undoubtedly share an edge with one another.”

Furthermore, there is no strong evidence that graph measures focusing on connectivity of individual voxels within larger areas will be distorted due to a misrepresentation of areas as a function of the voxels they contain. Nor is there any reason to believe that measures describing global properties of the graph will be distorted due to a misrepresentation of areas as a function of the voxels they contain. No matter how the putative functional area is represented, subsections of brain tissue within the functional area have very different properties. When treating these supposed functionally homogenous “units” as a single node in a functional brain network, researchers will fail to see different properties in different subregions of the functional area itself. Thus, the variation in the properties of voxels within functional areas is still worth investigating. In fact, by using a voxel-wise approach, it is possible to objectively discover functional areal distinctions of varying sizes in individual subjects, without having to resort to amassing an arbitrary set of pre-existing traditional fMRI studies—a process inherently prone to experimenter bias and error—in order to define putative functional areas. Furthermore, the voxel-wise approach allows for putative functional areas to change across people, conditions, states, and time. The meta-analytic approach assumes that the functional areas are pre-existing and do not change.

**Figure 4:**
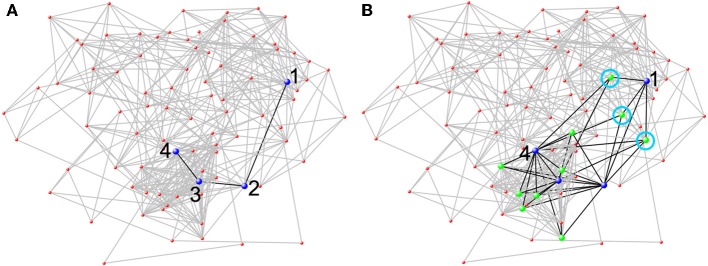
**Network analysis from a single subject at rest based on a 90 node AAL parcellation.** The 90 node network was chosen, because it is easily amenable to being visually represented as a whole. The figure shows how conclusions from network analyses that do not use all brain nodes could dramatically differ from a whole-brain analysis. **(A)** When only considering the blue nodes (neglecting the rest of the network), there is limited connectivity between the four nodes (black lines). To get from Node 1 to Node 4 it requires three steps. **(B)** However, when the entire network is included, other nodes (green nodes) that connect to two or more blue nodes can be seen. Through the green nodes, new pathways between the blue nodes are evident. There are now three different pathways through a green node (each one is circled in light blue) such that Node 1 can reach Node 4 in two steps.

Using smaller spheres or cubes to represent larger functional areas or merely choosing a partial set of brain areas for network analysis will fail to incorporate all brain space with detectable signal into network analysis. Consequently, such approaches likely misrepresent local and global network measures and fail to reap the distinctive benefits of a network-based approach. Connectivity can be assessed between all brain areas simultaneously using network science. This is an incredible leap forward in neuroscientific research. Prior to 2005, researchers were limited to evaluating connectivity between a few select areas at a time. While valuable, such analyses do not allow researchers to examine how any particular area fits into overall network organization. The clear benefits of examining how a particular area fits into overall network organization can be illustrated by examining the efficiency of information flow in a network (see Figure 4). When examining the connectivity exclusively between 4 different brain areas of a larger network, one would have an idea how those four areas communicated with each other, but it would be impossible to know how the efficiency of other brain areas provide alternate paths of information transfer. Figure 4 shows how conclusions from connectivity analyses for a select group of regions could dramatically differ from a whole-brain analysis. Each portion of neglected brain space could potentially contribute to several functions of the network as a whole. The fact that all possible connections are included in a network analysis ensures that both whole-brain and region-to-region connectivity can be evaluated accurately. For, all nodal definition schemes should allow for the meaningful interpretation of both local and global metrics.

All approaches that define putative ROIs from functional activation studies are fundamentally restricted by the current availability and breadth of imaging studies (Wig et al., 2011, 2013). The set of tasks used to predefine putative functional areas varies significantly from researcher to researcher. For instance, Wang et al. (2011) used 5 different tasks, including: error-processing, default-mode, memory, language and sensorimotor. In contrast, Power et al. (2011) used 9 different tasks, including: button-pushing, verb generation, reading, sustained task-induced deactivations, transient task-induced deactivations, sustained task block activations, on-cue task block activations, error commission, and memory retrieval. Not only is it difficult, or perhaps impossible, to compare such different brain networks (Honey et al., 2009; Kaiser, 2011), but also there is no reason to assume that any one particular set of tasks is better than the other. The set of tasks used by Wang et al. (2011) do not include any spatially overlapping ROIs, nullifying the problem of having to either randomly (and thus arbitrarily) delete encroaching ROIs or spatially average certain ROIs due to encroachment. However, the set of tasks used by Power et al. (2011) is more comprehensive, likely producing a slightly fuller depiction of brain activation foci. Still, only incorporating 9 different tasks to define all functional areas in the brain can only produce an incomplete description of the location and extent of all putative functional areas, especially since so many identified ROIs were either randomly deleted or spatially averaged together. There is no neuroscientific reason to assume that the peaks of activation ascertained by analyzing data from 9 kinds of tasks will be applicable to all types of tasks. No set of tasks should be considered complete or comprehensive.

Importantly, as more tasks are incorporated into the meta-analysis, it becomes more and more likely that putative functional areas overlap. For instance, by only incorporating 9 different tasks in a meta-analysis to define nodes, Power et al. (2011) had to spatially average 171 putative ROIs (represented as spheres on peak activity coordinates), because they were encroaching upon one another. Presumably, a larger, more complete corpus of task-evoked fMRI data will contain many more overlapping nodes. And, as one progresses toward including a more “complete” set of task-evoked fMRI data to identify putative functional areas, then all areas of the brain will collectively become activation foci. As more task-evoked data is compiled, more and more spheres will be spatially encroaching on one another, requiring researchers to randomly delete encroaching ROIs or spatially average encroaching ROIs. Both options will fail to accurately represent the network.

In addition to the issues of individual variability, we contend that the functional activation approach cannot be applied to interventional studies, longitudinal observational studies, or studies that examine the brain networks of populations with distinct brain physiology. Patterns of neural activity in response to various task demands change considerably across the lifespan (Stiles, 2008; Power et al., 2010; Vogel et al., 2010), and the literature is full of studies showing different brain activation patterns across patient populations. The peak activity coordinates of putative functional areas identified by this approach in subjects within a certain age group or diagnostic category may not be transferrable to subjects in a different age group or diagnostic category.

In contrast to functional activation approaches, using a voxel-wise approach allows for a model-free, unbiased examination of both inter-regional and intra-regional connectivity. Consequently, the voxel-wise method has the potential to reveal new information about network organization, without relying on an arbitrary set of preexisting traditional task-related fMRI studies. Network science has tremendous promise to tell us something new about the functional nature of the brain. However, traditional fMRI studies identify a linear association between task and brain activity. If network science studies of the brain are limited by findings from traditional fMRI, then will be able to provide little insight beyond what has already been discovered. The network science approach holds tremendous promise for identifying new relationships in the brain, but this is not possible if we are limited to what previous non-network fMRI analyses have already demonstrated (Telesford et al., 2011).

## Conclusion

This manuscript has examined the predominate schemes that are currently used for defining nodes in functional brain networks: voxel-wise, anatomical atlas-based, and functional activation meta-analytic. We argue that voxel-wise networks are the most likely to result in new discoveries of unknown brain properties. The other methods are limited by *a priori* knowledge, and therefore, limit our ability to make new discoveries. Voxel-wise networks have the resolution to allow for the identification of key network nodes that are encompassed within larger anatomical regions. Voxel-wise networks also allow for the discovery of key nodes that are not located within a putative functional unit. Overall, the voxel-wise method is data-driven and allows for discoveries that cannot be achieved by other methods. Undoubtedly there are limitations to the voxel-wise approach, but we believe that the evidence for these potential limitations is currently poor and that the strengths of the voxel-wise approach outweigh the potential weaknesses.

There is a fundamental difference in the underlying accepted presuppositions among those who do brain network analyses (complexity theory) and those who do functional brain mapping (reductionism). Applying *a priori* information from prior research based on anatomy or brain activation research limits brain network analyses to the discoveries made in these fields. Such methods will surely provide support for the past studies of localization of function as the nodes are based on such research. If the goal is simply to replicate past findings using a new method, then much less effort should be directed toward this goal. If the intent is to discover new principles and organization of the human brain, then researchers should not be tethered to the outcomes of past work. If network science studies do not replicate past fMRI findings or do not corroborate structure-function relationships that have been tied to brain anatomy, then either we are discovering new things about how the brain works or we are wrong. We should explore these two possibilities and recognize that all or our models are wrong and that the new ideas coming from network science are moving us closer to truth about the brain. The litmus test is going to be whether brain network research enables us to better understand human behavior, brain diseases, clinical treatments, and the mind. The test should not be whether brain networks reinforce what we already believe about the brain, because at this stage, our understanding of the human brain is rather trivial.

Why not use an approach that does not include *a priori* assumptions about functional subunits of the brain? We have heard the argument that without *a priori* assumptions, we cannot understand and interpret findings. Many ask what the findings mean in relation to all the previous neuroscience studies. We would suggest responding that one should not bias his/her results with concepts that may hold back the field. Why not see if network science can fundamentally alter our view of the brain and brain function, because it is a fundamentally new way of thinking. It does not assume certain tissues in the brain are static functional units. Rather, it allows for a dynamic brain, able to perform complex functions, which are emergent network features of the system. We hold that the “static functional unit” view is more wrong than the non-biased dynamic view. We acknowledge that the latter view (i.e., a voxel-wise approach) is also flawed, but let's not handcuff these new techniques with past methodologies.

We contend that no nodal parcellation scheme has been developed that is capable of providing valuable information beyond what the voxel-wise approach has already shown. Additionally, because the voxel-wise approach does not require implementing any *a priori* assumptions regarding what constitutes the “right” node, the approach is fundamentally unbiased. Therefore, the approach allows the data to speak for itself. Though we have argued that the voxel-wise method for defining nodes in functional brain networks is a better method than the other available options, the “best” method to be used remains an open question deserving debate and additional research. However, rather than being debated, it is more common for peer reviewers to reject manuscripts that do not use the method that they deem appropriate. This review was meant to be a step forward in fostering discussion. It is critical that we acknowledge the fact that the absolutely correct parcellation scheme remains an enigma, and it is possible that multiple different parcellation schemes developed in the future will be valuable and meaningful in elucidating different network properties.

### Conflict of interest statement

The authors declare that the research was conducted in the absence of any commercial or financial relationships that could be construed as a potential conflict of interest.
