# Supplementary material for: Defining nodes in complex brain networks
Source: Front Comput Neurosci. 2013 Nov 22;7:169. doi: 10.3389/fncom.2013.00169 (PMC3837224; doi:10.3389/fncom.2013.00169)
Supplement: Supplementary file 1 [file DataSheet1.PDF]

Voxel-Wise Parcellations

AUTHORS	YEAR	TITLE
• Burdette, J. H., Laurienti, P. J., Espeland, M. A., Morgan, A., Telesford, Q., Vechlekar, C. D., Hayasaka, S.H., Jennings, J.M., Katula, J.A., Kraft, R.A., & Rejeski, W. J.	2010	Using network science to evaluate exercise-associated brain changes in older adults.
• Chen, Gang, Ward, B. D., Xie, C., Li, W., Chen, G., Goveas, J. S., Antuono, P.G. & Li, S.H.	2012	A clustering-based method to detect functional connectivity differences.
• Cole, M. W., Pathak, S., & Schneider, W.	2010	Identifying the brain's most globally connected regions.
• De Pasquale, F., Sabatini, U., Della Penna, S., Sestieri, C., Caravasso, C. F., Formisano, R., & Péran, P.	2013	The connectivity of functional cores reveals different degrees of segregation and integration in the brain at rest.
• Dey, S., Rao, A. R., & Shah, M.	2012	Exploiting the brain's network structure in identifying ADHD subjects.
• Eguíluz, V. M., Chialvo, D. R., Cecchi, G. A., Baliki, M., & Apkarian, A. V.	2005	Scale-free brain functional networks.
• Ferrarini, L., Veer, I. M., van Lew, B., Oei, N. Y. L., van Buchem, M. A., Reiber, J. H. C., Rombouts, S.A.R.B. & Miles, J.	2011	Non-parametric model selection for subject-specific topological organization of resting-state functional connectivity.
• Fransson, P., Aden, U., Blennow, M., & Lagercrantz, H.	2011	The functional architecture of the infant brain as revealed by resting-state fMRI.
• Gallos, L. K., Makse, H. A., & Sigman, M.	2012	A small world of weak ties provides optimal global integration of self-similar modules in functional brain networks.
• Gallos, L. K., Sigman, M., & Makse, H. A.	2012	The conundrum of functional brain networks: small-world efficiency or fractal modularity.
• Joyce, K. E., Hayasaka, S., & Laurienti, P. J.	2013	The human functional brain network demonstrates structural and dynamical resilience to targeted attack.
• Joyce, K. E., Laurienti, P. J., Burdette, J. H., & Hayasaka, S.	2010	A new measure of centrality for brain networks.
• Keilholz, S. D., Magnuson, M., & Thompson, G.	2010	Evaluation of data-driven network analysis approaches for functional connectivity MRI.
• Kinreich, S., Intrator, N., & Hendler, T.	2011	Functional cliques in the amygdala and related brain networks driven by fear assessment acquired during movie viewing.
• Liu, J., Qin, W., Yuan, K., Li, J., Wang, W., Li, Q., Wang, Y., Sun, J., von Deneen, K.M., Liu, Y. & Tian, J.	2011	Interaction between dysfunctional connectivity at rest and heroin cues-induced brain responses in male abstinent heroin-dependent individuals.
• Matthäus, F., Schmidt, J.-P., Banerjee, A., Schulze, T. G., Demirakca, T., & Diener, C.	2012	Effects of age on the structure of functional connectivity networks during episodic and working memory demand.
• Moussa, Malaak N, Steen, M. R., Laurienti, P. J., & Hayasaka, S.	2012	Consistency of network modules in resting-state FMRI connectome data.
• Moussa, Malaak Nasser, Vechlekar, C. D., Burdette, J. H., Steen, M. R., Hugenschmidt, C. E., & Laurienti, P. J.	2011	Changes in cognitive state alter human functional brain networks.
• Telesford, Q. K., Morgan, A. R., Hayasaka, S., Simpson, S. L., Barret, W., Kraft, R. A., Mozolic, J.L. & Laurienti, P. J.	2010	Reproducibility of graph metrics in FMRI networks.
• Tomasi, D., & Volkow, N. D.	2012	Aging and functional brain networks.
• Tomasi, D., & Volkow, N. D.	2010	Functional connectivity density mapping.
• Tomasi, D., & Volkow, N. D.	2011	Functional connectivity hubs in the human brain.
• Tomasi, D., & Volkow, N. D.	2011	Association between functional connectivity hubs and brain networks.
• Valencia, M., Pastor, M. A., Fernández-Seara, M. A., Artieda, J., Martinerie, J., & Chavez, M.	2009	Complex modular structure of large-scale brain networks.
• Van den Heuvel, M P, Stam, C. J., Boersma, M., & Hulshoff Pol, H. E.	2008	Small-world and scale-free organization of voxel-based resting-state functional connectivity in the human brain.
• Van den Heuvel, Martijn P, Stam, C. J., Kahn, R. S., & Hulshoff Pol, H. E.	2009	Efficiency of functional brain networks and intellectual performance.
• Voss, M. W., Prakash, R. S., Erickson, K. I., Basak, C., Chaddock, L., Kim, J. S., Alves, H., Heo, S., Szabo, A.N., White, S.M., Wojcicki, T.R., Mailey, E.L., Gothe, N., Olson, E.A., McAuley, E. & Kramer, A. F.	2010	Plasticity of brain networks in a randomized intervention trial of exercise training in older adults.
• Yuan, K., Qin, W., Liu, J., Guo, Q., Dong, M., Sun, J., Zhang, Y., Liu, P., Wang, W., Wang, Y., Li, Q., Yang, W., von Deneen, K.M., Gold, M.S.,	2010	Altered small-world brain functional networks and duration of heroin use in male abstinent heroin-dependent individuals.

Liu, Y. & Tian, J.

Structural Anatomical Atlas-Based Parcellations

AUTHORS	YEAR	TITLE
• Achard, S., & Bullmore, E.	2007	Efficiency and cost of economical brain functional networks.
• Achard, S., Delon-Martin, C., Vértes, P. E., Renard, F., Schenck, M., Schneider, F., Heinrich, C., Kremer, S. & Bullmore, E. T.	2012	Hubs of brain functional networks are radically reorganized in comatose patients.
• Achard, S., Salvador, R., Whitcher, B., Suckling, J., & Bullmore, E.	2006	A resilient, low-frequency, small-world human brain functional network with highly connected association cortical hubs.
• Alexander-Bloch, A. F., Gogtay, N., Meunier, D., Birn, R., Clasen, L., Lalonde, F., Lenroot, R., Gied, J. & Bullmore, E. T.	2010	Disrupted modularity and local connectivity of brain functional networks in childhood-onset schizophrenia.
• Alexander-Bloch, A. F., Vértes, P. E., Stidd, R., Lalonde, F., Clasen, L., Rapoport, J., Giedd, J., Bullmore, E.T. & Gogtay, N.	2013	The anatomical distance of functional connections predicts brain network topology in health and schizophrenia.
• Alexander-Bloch, A., Lambiotte, R., Roberts, B., Giedd, J., Gogtay, N., & Bullmore, E.	2012	The discovery of population differences in network community structure: new methods and applications to brain functional networks in schizophrenia.
• Balenzuela, P., Chernomoretz, A., Fraiman, D., Cifre, I., Sitges, C., Montoya, P., & Chialvo, D. R.	2010	Modular organization of brain resting state networks in chronic back pain patients.
• Bassett, D. S., Wymbs, N. F., Porter, M. A., Mucha, P. J., Carlson, J. M., & Grafton, S. T.	2011	Dynamic reconfiguration of human brain networks during learning.
• Bohr, I. J., Kenny, E., Blamire, A., O'Brien, J. T., Thomas, A. J., Richardson, J., & Kaiser, M.	2012	Resting-state functional connectivity in late-life depression: higher global connectivity and more long distance connections.
• Braun, U., Plichta, M. M., Esslinger, C., Sauer, C., Haddad, L., Grimm, O., Mier, D., Mohnke, S., Heinz, A., Erk, S., Walter, H., Seiferth, N., Kirsch, P. & Meyer-Lindenberg, A.	2012	Test-retest reliability of resting-state connectivity network characteristics using fMRI and graph theoretical measures.
• Bruno, J., Hosseini, S. M. H., & Kesler, S.	2012	Altered resting state functional brain network topology in chemotherapy-treated breast cancer survivors.
• Cabral, J., Hugues, E., Kringelbach, M. L., & Deco, G.	2012	Modeling the outcome of structural disconnection on resting-state functional connectivity.
• Casanova, R., Whitlow, C. T., Wagner, B., Espeland, M. A., & Maldjian, J. A.	2012	Combining graph and machine learning methods to analyze differences in functional connectivity across sex.
• Chen, Gang, Ward, B. D., Xie, C., Li, W., Wu, Z., Jones, J. L., Franczak, M., Antuono, P. & Li, S.-J.	2011	Classification of Alzheimer disease, mild cognitive impairment, and normal cognitive status with large-scale network analysis based on resting-state functional MR imaging.
• Chen, Guangyu, Chen, G., Xie, C., & Li, S.-J.	2011	Negative functional connectivity and its dependence on the shortest path length of positive network in the resting-state human brain.
• Ciftçi, K.	2011	Minimum spanning tree reflects the alterations of the default mode network during Alzheimer's disease.
• Cocchi, L., Bramati, I. E., Zalesky, A., Furukawa, E., Fontenelle, L. F., Moll, J., Tripp, G., Mattos, P.	2012	Altered functional brain connectivity in a non-clinical sample of young adults with attention-deficit/hyperactivity disorder.
• Davis, F. C., Knodt, A. R., Sporns, O., Lahey, B. B., Zald, D. H., Brigidi, B. D., & Hariri, A. R.	2013	Impulsivity and the modular organization of resting-state neural networks.
• Feng, Y., Bai, L., Ren, Y., Wang, H., Liu, Z., Zhang, W., & Tian, J.	2011	Investigation of the large-scale functional brain networks modulated by acupuncture.
• Ferrarini, L., Veer, I. M., Baerends, E., van Tol, M.-J., Renken, R. J., van der Wee, N. J. A., Veltman, D.J., Aleman, A., Zitman, F.G., Penninx, B.W.J.H, van Buchem, M.A., Reiber, J.H.C., Rombouts, S.A.R.B. & Milles, J.	2009	Hierarchical functional modularity in the resting-state human brain.
• Fornito, A., Yoon, J., Zalesky, A., Bullmore, E. T., & Carter, C. S.	2011	General and specific functional connectivity disturbances in first-episode schizophrenia during cognitive control performance.
• Fornito, A., Zalesky, A., Bassett, D. S., Meunier, D., Ellison-Wright, I., Yücel, M., Wood, S.J., Shaw, K., O'Connor, J., Nertney, D., Mowry, B.J., Pantelis, C. & Bullmore, E. T.	2011	Genetic influences on cost-efficient organization of human cortical functional networks.

• Fornito, A., Zalesky, A., & Bullmore, E. T.	2010	Network scaling effects in graph analytic studies of human resting-state FMRI data.
• Ginestet, C. E., & Simmons, A.	2011	Statistical parametric network analysis of functional connectivity dynamics during a working memory task.
• Gleiser, P. M., & Spoormaker, V. I.	2010	Modeling hierarchical structure in functional brain networks. Philosophical transactions.
• Gratton, C., Nomura, E. M., Pérez, F., & D'Esposito, M.	2012	Focal brain lesions to critical locations cause widespread disruption of the modular organization of the brain.
• Guo, H., Cao, X., Liu, Z., Li, H., Chen, J., & Zhang, K.	2012	Machine learning classifier using abnormal brain network topological metrics in major depressive disorder.
• Hartman, D., Hlinka, J., Palus, M., Mantini, D., & Corbetta, M.	2011	The role of nonlinearity in computing graph-theoretical properties of resting-state functional magnetic resonance imaging brain networks.
• He, Y., Wang, J., Wang, L., Chen, Z. J., Yan, C., Yang, H., … Evans, A. C.	2009	Uncovering intrinsic modular organization of spontaneous brain activity in humans.
• Honey, C. J., Sporns, O., Cammoun, L., Gigandet, X., Thiran, J. P., Meuli, R., & Hagmann, P.	2009	Predicting human resting-state functional connectivity from structural connectivity.
• Hosseini, S. M. H., Hoeft, F., & Kesler, S. R.	2012	GAT: a graph-theoretical analysis toolbox for analyzing between-group differences in large-scale structural and functional brain networks.
• Hsu, T.-W., Wu, C. W., Cheng, Y.-F., Chen, H.-L., Lu, C.-H., Cho, K.-H., Lin, W.C. & Lin, C.-P.	2012	Impaired small-world network efficiency and dynamic functional distribution in patients with cirrhosis.
• Jin, C., Gao, C., Chen, C., Ma, S., Netra, R., Wang, Y., Zhang, M. & Li, D.	2011	A preliminary study of the dysregulation of the resting networks in first-episode medication-naive adolescent depression.
• Liang, X., Wang, J., Yan, C., Shu, N., Xu, K., Gong, G., & He, Y.	2012	Effects of different correlation metrics and preprocessing factors on small-world brain functional networks: a resting-state functional MRI study.
• Liao, W., Zhang, Z., Pan, Z., Mantini, D., Ding, J., Duan, X., Lu, G. & Chen, H.	2010	Altered functional connectivity and small-world in mesial temporal lobe epilepsy.
• Liu, B., Chen, J., Wang, J., Liu, X., Duan, X., Shang, X., Long, Y., Chen, Z., Li, X., Huang, Y. & He, Y.	2012	Altered small-world efficiency of brain functional networks in acupuncture at ST36: a functional MRI study.
• Liu, J., Qin, W., Nan, J., Li, J., Yuan, K., Zhao, L., Zeng, F., Sun, J., Yu, D., Dong, M., Liu, P., Von Deneen, K.M., Gong, Q., Liang, F. & Tian, J.	2011	Gender-related differences in the dysfunctional resting networks of migraine suffers.
• Liu, J., Zhao, L., Li, G., Xiong, S., Nan, J., Li, J., Yuan, K., von Deneen, K.M., Liang, F., Qin, W. & Tian, J.	2012	Hierarchical alteration of brain structural and functional networks in female migraine sufferers.
• Liu, Y., Liang, M., Zhou, Y., He, Y., Hao, Y., Song, M., Yu, C., Liu, H., Liu, Z. & Jiang, T.	2008	Disrupted small-world networks in schizophrenia.
• Liu, Z., Zhang, Y., Yan, H., Bai, L., Dai, R., Wei, W., Zhong, C., Xue, T., Wang, H., Feng, Y., You, Y., Zhang, X. & Tian, J.	2012	Altered topological patterns of brain networks in mild cognitive impairment and Alzheimer's disease: a resting-state fMRI study.
• Lord, L.-D., Allen, P., Expert, P., Howes, O., Lambiotte, R., McGuire, P., Bose, S., Hyde, S. & Turkheimer, F. E.	2011	Characterization of the anterior cingulate's role in the at-risk mental state using graph theory.
• Loui, P., Zamm, A., & Schlaug, G.	2012	Enhanced functional networks in absolute pitch.
• Lynall, M.-E., Bassett, D. S., Kerwin, R., McKenna, P. J., Kitzbichler, M., Muller, U., & Bullmore, E.	2010	Functional connectivity and brain networks in schizophrenia.
• Meunier, D., Achard, S., Morcom, A., & Bullmore, E.	2009	Age-related changes in modular organization of human brain functional networks.
• Meunier, D., Lambiotte, R., Fornito, A., Ersche, K. D., & Bullmore, E. T.	2009	Hierarchical modularity in human brain functional networks.
• Mirzasoleiman, B., & Jalili, M.	2011	Failure tolerance of motif structure in biological networks.
• Park, B., Kim, J. I., Lee, D., Jeong, S.-O., Lee, J. D., & Park, H.-J.	2012	Are brain networks stable during a 24-hour period?
• Park, C., Boudrias, M.-H., Rossiter, H., & Ward, N. S.	2012	Age-related changes in the topological architecture of the brain during hand grip.
• Rubinov, M., & Sporns, O.	2011	Weight-conserving characterization of complex functional brain networks.
• Sanz-Arigita, E. J., Schoonheim, M. M., Damoiseaux, J. S., Rombouts, S. A. R. B., Maris, E., Barkhof, F., Scheltens, P. & Stam, C. J.	2010	Loss of "small-world" networks in Alzheimer's disease: graph analysis of FMRI resting-state functional connectivity.
• Schwarz, A. J., & McGonigle, J	2011	Negative edges and soft thresholding in complex network analysis of resting state functional connectivity data.
• Sheppard, John Patrick, Wang, J.-P., & Wong, P. C. M.	2012	Large-scale cortical network properties predict future sound-to-word learning success.

• Sheppard, John P, Wang, J.-P., & Wong, P. C. M.	2011	Large-scale cortical functional organization and speech perception across the lifespan
• Simpson, S. L., Moussa, M. N., & Laurienti, P. J.	2012	An exponential random graph modeling approach to creating group-based representative whole-brain connectivity networks.
• Skidmore, F., Korenkevych, D., Liu, Y., He, G., Bullmore, E., & Pardalos, P. M.	2011	Connectivity brain networks based on wavelet correlation analysis in Parkinson fMRI data.
• Spoormaker, V. I., Schröter, M. S., Gleiser, P. M., Andrade, K. C., Dresler, M., Wehrle, R., Samann, P.G. & Czisch, M.	2010	Development of a Large-Scale Functional Brain Network during Human Non-Rapid Eye Movement Sleep.
• Supekar, K., Menon, V., Rubin, D., Musen, M., & Greicius, M. D.	2008	Network analysis of intrinsic functional brain connectivity in Alzheimer’s disease.
• Supekar, K., Musen, M., & Menon, V.	2009	Development of large-scale functional brain networks in children.
• Tao, H., Guo, S., Ge, T., Kendrick, K. M., Xue, Z., Liu, Z., & Feng, J.	2013	Depression uncouples brain hate circuit.
• Tian, L., Wang, J., Yan, C., & He, Y.	2011	Hemisphere- and gender-related differences in small-world brain networks: a resting-state functional MRI study.
• Vaessen, M. J., Braakman, H. M. H., Heerink, J. S., Jansen, J. F. A., Debeij-van Hall, M. H. J. A., Hofman, P. A. M., Aldenkamp, A.P. & Backes, W. H.	2012	Abnormal Modular Organization of Functional Networks in Cognitively Impaired Children with Frontal Lobe Epilepsy.
• Van den Heuvel, Martijn P, & Sporns, O.	2011	Rich-club organization of the human connectome.
• Varoquaux, G., Gramfort, A., Poline, J. B., & Thirion, B.	2012	Markov models for fMRI correlation structure: Is brain functional connectivity small world, or decomposable into networks?
• Vértes, P. E., Alexander-Bloch, A. F., Gogtay, N., Giedd, J. N., Rapoport, J. L., & Bullmore, E. T.	2012	Simple models of human brain functional networks.
• Vértes, P. E., Nicol, R. M., Chapman, S. C., Watkins, N. W., Robertson, D. A., & Bullmore, E. T.	2011	Topological isomorphisms of human brain and financial market networks.
• Wang, J., Wang, L., Zang, Y., Yang, H., Tang, H., Gong, Q., … He, Y.	2009	Parcellation-dependent small-world brain functional networks: a resting-state fMRI study.
• Wang, L., Zhu, C., He, Y., Zang, Y., Cao, Q., Zhang, H., Zhong, Q. & Wang, Y.	2009	Altered small-world brain functional networks in children with attention-deficit/hyperactivity disorder.
• Wang, L., Li, Y., Metzak, P., He, Y., & Woodward, T. S.	2010	Age-related changes in topological patterns of large-scale brain functional networks during memory encoding and recognition.
• Wang, Z., Liu, J., Zhong, N., Qin, Y., Zhou, H., & Li, K.	2012	Changes in the brain intrinsic organization in both on-task state and post-task resting state.
• Wee, C.-Y., Yap, P.-T., Denny, K., Browndyke, J. N., Potter, G. G., Welsh-Bohmer, K. A., Wang, L. & Shen, D.	2012	Resting-state multi-spectrum functional connectivity networks for identification of MCI patients.
• Wee, C.-Y., Yap, P.-T., Zhang, D., Wang, L., & Shen, D.	2012	Constrained sparse functional connectivity networks for MCI classification.
• Whitlow, C. T., Casanova, R., & Maldjian, J. A.	2011	Effect of resting-state functional MR imaging duration on stability of graph theory metrics of brain network connectivity.
• Wozniak, J. R., Mueller, B. A., Bell, C. J., Muetzel, R. L., Hoecker, H. L., Boys, C. J., & Lim, K. O.	2013	Global functional connectivity abnormalities in children with fetal alcohol spectrum disorders.
• Wu, K., Taki, Y., Sato, K., Hashizume, H., Sassa, Y., Takeuchi, H., … Fukuda, H.	2013	Topological organization of functional brain networks in healthy children: differences in relation to age, sex, and intelligence.
• Wylie, K. P., Rojas, D. C., Tanabe, J., Martin, L. F., & Tregellas, J. R.	2012	Nicotine increases brain functional network efficiency.
• Xue, S., Tang, Y.-Y., & Posner, M. I.	2011	Short-term meditation increases network efficiency of the anterior cingulate cortex.
• Yan, C., & He, Y.	2011	Driving and driven architectures of directed small-world human brain functional networks.
• Yu, D.	2013	Additional brain functional network in adults with attention-deficit/hyperactivity disorder: a phase synchrony analysis.
• Zalesky, A., Fornito, A., & Bullmore, E. T.	2010	Network-based statistic: identifying differences in brain networks.
• Zhang, J., Wang, J., Wu, Q., Kuang, W., Huang, X., He, Y., & Gong, Q.	2011	Disrupted brain connectivity networks in drug-naive, first-episode major depressive disorder.
• Zhang, Z., Liao, W., Chen, H., Mantini, D., Ding, J.-R., Xu, Q., Wang, Z., Yuan, C., Chen, G., Jiao, Q. & Lu, G.	2011	Altered functional-structural coupling of large-scale brain networks in idiopathic generalized epilepsy.
• Zhao, Xiaohu, Liu, Y., Wang, X., Liu, B., Xi, Q., Guo, Q., Jiang, H., Jiang, T. & Wang, P.	2012	Disrupted small-world brain networks in moderate Alzheimer’s disease: a resting-state FMRI study.
• Zhao, Xiao-hu, Wang, X., Xi, Q., Jiang, H., Guo, Q., Liu, Y., & Wang, P.	2012	Small-worldness of functional networks in Alzheimer’s disease.

Functional Activation Meta-Analytic Parcellations

AUTHORS	YEAR	TITLE
• Barttfeld, P., Wicker, B., Cukier, S., Navarta, S., Lew, S., Leiguarda, R., & Sigman, M.	2012	State-dependent changes of connectivity patterns and functional brain network topology in autism spectrum disorder.
• Caeyenberghs, K., Leemans, A., Heitger, M. H., Leunissen, I., Dhollander, T., Sunaert, S., Dupont, P. & Swinnen, S. P.	2012	Graph analysis of functional brain networks for cognitive control of action in traumatic brain injury.
• Cheng, L., Wu, Z., Fu, Y., Miao, F., Sun, J., & Tong, S.	2012	Reorganization of functional brain networks during the recovery of stroke: a functional MRI study.
• Dosenbach, N. U. F., Fair, D. A., Cohen, A. L., Schlaggar, B. L., & Petersen, S. E.	2008	A dual-networks architecture of top-down control.
• Dosenbach, N. U. F., Fair, D. A., Miezin, F. M., Cohen, A. L., Wenger, K. K., Dosenbach, R. A. T., Fox, M.D., Snyder, A.Z., Vincent, J.L., Raichle, M.E., Schlagger, B.L. & Petersen, S. E.	2007	Distinct brain networks for adaptive and stable task control in humans.
• Fair, D. A., Cohen, A. L., Power, J. D., Dosenbach, N. U. F., Church, J. A., Miezin, F. M., Schlagger, B.L. & Petersen, S. E.	2009	Functional brain networks develop from a “local to distributed” organization.
• Fair, D. A., Dosenbach, N. U. F., Church, J. A., Cohen, A. L., Brahmbhatt, S., Miezin, F. M., Barch, D.M. , Raichle, M.E., Peterson, S.E. & Schlaggar, B. L.	2007	Development of distinct control networks through segregation and integration.
• He, B. J.	2011	Scale-free properties of the functional magnetic resonance imaging signal during rest and task.
• Heitger, M. H., Ronsse, R., Dhollander, T., Dupont, P., Caeyenberghs, K., & Swinnen, S. P.	2012	Motor learning-induced changes in functional brain connectivity as revealed by means of graph-theoretical network analysis.
• Hwang, K., Hallquist, M. N., & Luna, B.	2012	The Development of Hub Architecture in the Human Functional Brain Network.
• Minati, L., Grisoli, M., Seth, A. K., & Critchley, H. D.	2012	Decision-making under risk: a graph-based network analysis using functional MRI.
• Nomura, E. M., Gratton, C., Visser, R. M., Kayser, A., Perez, F., & D’Esposito, M.	2010	Double dissociation of two cognitive control networks in patients with focal brain lesions.
• Rish, I., Cecchi, G., Thyreau, B., Thirion, B., Plaze, M., Paillere-Martinot, M. L., Martelli, C., Martinot, J.L. & Poline, J.-B.	2013	Schizophrenia as a network disease: disruption of emergent brain function in patients with auditory hallucinations.
• Stevens, A. A., Tappon, S. C., Garg, A., & Fair, D. A.	2012	Functional brain network modularity captures inter- and intra-individual variation in working memory capacity.
• Wang, J.-H., Zuo, X.-N., Gohel, S., Milham, M. P., Biswal, B. B., & He, Y.	2011	Graph theoretical analysis of functional brain networks: test-retest evaluation on short- and long-term resting-state functional MRI data.
• Yan, X., Kelley, S., Goldberg, M., & Biswal, B. B.	2011	Detecting overlapped functional clusters in resting state fMRI with Connected Iterative Scan: a graph theory based clustering algorithm.
• Zhang, T., Wang, J., Yang, Y., Wu, Q., Li, B., Chen, L., Yue, Q., Tang, H., Yan, C., Lui, S., Huang, X., Chan, R.C.K., Zang, Y., He, Y. & Gong, Q.	2011	Abnormal small-world architecture of top-down control networks in obsessive-compulsive disorder.

Multiple Parcellations

AUTHORS	YEAR	TITLE
• Buckner, R. L., Sepulcre, J., Talukdar, T., Krienen, F. M., Liu, H., Hedden, T., Hedden, T., Andrews-Hanna, J.R., Sperling, R.A. & Johnson, K. A.	2009	Cortical hubs revealed by intrinsic functional connectivity: mapping, assessment of stability, and relation to Alzheimer’s disease.
• Cole, M. W., Yarkoni, T., Repovs, G., Anticevic, A., & Braver, T. S.	2012	Global connectivity of prefrontal cortex predicts cognitive control and intelligence.
• Hayasaka, S., & Laurienti, P. J.	2010	Comparison of characteristics between region-and voxel-based network analyses in resting-state fMRI data.
• Power JD, Cohen AL, Nelson SM, Wig GS, Barnes KA, Church JA, Vogel AC, Laumann TO, Miezin FM, Schlaggar BL, Petersen SE	2011	Functional network organization of the human brain.
• Schröter, M., Spoormaker, V., Schorer, A., Wohlschläger, A., Czisch, M., Kochs, E., Zimmer, C., Hemmer, B., Schneider, G., Jordan, D. & Ilg, R.	2012	Spatiotemporal reconfiguration of large-scale brain functional networks during propofol-induced loss of consciousness.

Non-Traditional Parcellations

AUTHORS	YEAR	TITLE
• Brown, J. A., Rudie, J. D., Bandrowski, A., Van Horn, J. D., & Bookheimer, S. Y.	2012	The UCLA multimodal connectivity database: a web-based platform for brain connectivity matrix sharing and analysis.
• Ding, J.-R., Liao, W., Zhang, Z., Mantini, D., Xu, Q., Wu, G.-R., … Chen, H.	2011	Topological fractionation of resting-state networks.
• Ekman, M., Derrfuss, J., Tittgemeyer, M., & Fiebach, C. J.	2012	Predicting errors from reconfiguration patterns in human brain networks.
• Friston, K. J., Li, B., Daunizeau, J., & Stephan, K. E.	2011	Network discovery with DCM.
• He, H., Sui, J., Yu, Q., Turner, J. A., Ho, B.-C., Sponheim, S. R., … Calhoun, V. D.	2012	Altered small-world brain networks in schizophrenia patients during working memory performance.
• Jones, D. T., Vemuri, P., Murphy, M. C., Gunter, J. L., Senjem, M. L., Machulda, M. M., Przybelski, S.A., Gregg, B.E., Kantarci, K., Knopman, D.S., Boeve, B.F., Petersen, R.C. & Jack, C. R., Jr.	2012	Non-stationarity in the "resting brain's" modular architecture.
• Joseph, J. E., Swearingen, J. E., Clark, J. D., Benca, C. E., Collins, H. R., Corbly, C. R., Gathers, A.D. & Bhatt, R. S.	2012	The changing landscape of functional brain networks for face processing in typical development.
• Ma, S., Calhoun, V. D., Eichele, T., Du, W., & Adalı, T.	2012	Modulations of functional connectivity in the healthy and schizophrenia groups during task and rest.
• Maturana, P., & Batrancourt, B.	2011	A propositional representation model of anatomical and functional brain data.
• Mokhtari, F., & Hossein-Zadeh, G.-A.	2013	Decoding brain states using backward edge elimination and graph kernels in fMRI connectivity networks.
• Ren, Y., Bai, L., Feng, Y., Tian, J., & Li, K.	2010	Investigation of acupoint specificity by functional connectivity analysis based on graph theory.
• Seghier, M. L., & Friston, K. J.	2013	Network discovery with large DCMs.
• Spreng, R. N., Sepulcre, J., Turner, G. R., Stevens, W. D., & Schacter, D. L.	2013	Intrinsic architecture underlying the relations among the default, dorsal attention, and frontoparietal control networks of the human brain.
• Vejmelka, M., & Palus, M.	2010	Partitioning networks into clusters and residuals with average association.
• Wang, J., Zuo, X., Dai, Z., Xia, M., Zhao, Z., Zhao, X., Jia, J., Han, Y. & He, Y.	2013	Disrupted functional brain connectome in individuals at risk for Alzheimer's disease.
• Wang, L., Metzak, P. D., Honer, W. G., & Woodward, T. S.	2010	Impaired efficiency of functional networks underlying episodic memory-for-context in schizophrenia.
• Worbe, Y., Malherbe, C., Hartmann, A., Pélégrini-Issac, M., Messé, A., Vidailhet, M., … Benali, H.	2012	Functional immaturity of cortico-basal ganglia networks in Gilles de la Tourette syndrome.
• Yu, Q., Plis, S. M., Erhardt, E. B., Allen, E. A., Sui, J., Kiehl, K. A., … Calhoun, V. D.	2011	Modular Organization of Functional Network Connectivity in Healthy Controls and Patients with Schizophrenia during the Resting State.
• Yu, Q., Sui, J., Rachakonda, S., He, H., Gruner, W., Pearlson, G., … Calhoun, V. D.	2011	Altered topological properties of functional network connectivity in schizophrenia during resting state: a small-world brain network study.
• Yu, Q., Sui, J., Rachakonda, S., He, H., Pearlson, G., & Calhoun, V. D.	2011	Altered small-world brain networks in temporal lobe in patients with schizophrenia performing an auditory oddball task.
• Zhang, X., Tokoglu, F., Negishi, M., Arora, J., Winstanley, S., Spencer, D. D., & Constable, R. T.	2011	Social network theory applied to resting-state fMRI connectivity data in the identification of epilepsy networks with iterative feature selection.
• Zhou, J., Gennatas, E. D., Kramer, J. H., Miller, B. L., & Seeley, W. W.	2012	Predicting regional neurodegeneration from the healthy brain functional connectome.
